# Stretchable, Rehealable, Recyclable, and Reconfigurable Integrated Strain Sensor for Joint Motion and Respiration Monitoring

**DOI:** 10.34133/2021/9846036

**Published:** 2021-07-29

**Authors:** Chuanqian Shi, Zhanan Zou, Zepeng Lei, Pengcheng Zhu, Guohua Nie, Wei Zhang, Jianliang Xiao

**Affiliations:** ^1^Department of Mechanical Engineering, University of Colorado Boulder, Boulder, Colorado 80309, USA; ^2^School of Aerospace Engineering and Applied Mechanics, Tongji University, Shanghai 200092, China; ^3^Department of Chemistry, University of Colorado Boulder, Boulder, Colorado 80309, USA; ^4^School of Materials Science and Engineering, Beihang University, Beijing 100191, China

## Abstract

Cutting-edge technologies of stretchable, skin-mountable, and wearable electronics have attracted tremendous attention recently due to their very wide applications and promising performances. One direction of particular interest is to investigate novel properties in stretchable electronics by exploring multifunctional materials. Here, we report an integrated strain sensing system that is highly stretchable, rehealable, fully recyclable, and reconfigurable. This system consists of dynamic covalent thermoset polyimine as the moldable substrate and encapsulation, eutectic liquid metal alloy as the strain sensing unit and interconnects, and off-the-shelf chip components for measuring and magnifying functions. The device can be attached on different parts of the human body for accurately monitoring joint motion and respiration. Such a strain sensing system provides a reliable, economical, and ecofriendly solution to wearable technologies, with wide applications in health care, prosthetics, robotics, and biomedical devices.

## 1. Introduction

Soft and stretchable integrated electronic systems show superior mechanical compliance and deformability and thus can be applied in unusual places that are not possible for conventional rigid electronics, such as bioinspired imagers [1–3], biointegrated electronics for diagnosis and drug delivery [4–8], and electronic skins for health monitoring and virtual reality [9–13]. To achieve electronic performances comparable to the established semiconductor devices, off-the-shelf chip components were integrated with soft, stretchable substrates through mechanical designs that can effectively shield strains in brittle electronic components from the soft substrates experiencing large deformation [14–18]. For mechanical attributes, ultralow modulus and high stretchability were accomplished by using novel ideas in functional/hyperelastic materials [19–21], buckled metal traces [22–24], and liquid interconnects [25–33]. More recently, materials with self-healing capabilities have also been adopted in developing self-healable electronics to mimic natural skin [34–43]. In order to avoid surgical removal of medical implants, to protect security of hardware and data, or to reduce electronic waste [44–46], transient, degradable, and recyclable electronic systems were developed by using materials that can be physically eliminated within a specified period of time [13, 47–51].

As one representative type of stretchable electronics, stretchable strain sensing systems are widely used in robotics [28, 31], human motion detections [30–32, 41, 42], and health monitoring [17, 39, 51, 52]. In order to improve the performance of stretchable strain sensors, both solid and liquid sensing materials were explored, including silver nanowires, carbon nanotubes, gold nanoparticles, carbon grease, liquid metal, and ionic liquids [18, 28–33]. Among all these material choices, liquid metal provides the best combination of conductivity and deformability [26]. Serpentine structural designs were also adopted to improve stretchability [53–55]. Recently, more interesting properties were introduced into strain sensors, including self-healability, recyclability, and reconfigurability, by taking advantage of the progress in soft materials and chemistry [39–43, 50, 51]. These properties are beneficial to the long-term reliability, sustainability, and applicability of strain sensors and are not available in conventional substrate/encapsulation materials, like polydimethylsiloxane (PDMS). Hydrogels were used most often to realize self-healing, enabled by noncovalent hydrogen bonding [39–43]. However, dehydration in hydrogels can significantly degrade the performance of devices over time. Dynamic covalent thermosets can overcome the dehydration issue and can also achieve superior self-healable, recyclable, and reconfigurable capabilities. Furthermore, compared with noncovalent supramolecular materials, dynamic covalent thermosets provide more robust mechanical properties, and chemical and thermal stability [48, 56–58].

By utilizing the superior properties of liquid metal and dynamic covalent thermoset polyimine, we here introduce a high-performance integrated strain sensing system that is highly stretchable, rehealable, recyclable, and reconfigurable. The combination of liquid metal and polyimine is the key to achieving all these properties. As discussed above, the dynamic covalent chemistry of polyimine provides rehealing, recycling, and reconfiguring capabilities. As a high-performance conducting material, liquid metal is flowable; it does not add stresses to the device during deformation and reconfiguration of polyimine, leading to superior mechanical attributes to the device. Also, the flowability of liquid metal allows autonomous healing of electrical conductivity upon physical contact during rehealing of the device. It is the first strain sensing device that can achieve all these properties. These properties promise a cheap, robust, reliable, customizable, and sustainable wearable device, with positive impacts on economics, health care, and environment. This device consists of polyimine as the moldable substrate and encapsulation, eutectic liquid metal alloy as the strain sensing unit and interconnects, and off-the-shelf chip components for measuring and magnifying functions. The device can be attached on the knee, elbow, wrist, and finger joints for strain sensing and motion monitoring and can also be attached on the abdomen to accurately measure respiration cycles. When integrated with a light-emitting diode (LED), this device can provide real-time warning of excessive joint motions during training or other physical activities. Unlike a conventional rotary encoder strain sensor [59], such wearable devices are beneficial for health monitoring, due to their soft and stretchable characteristics. Moreover, this device can be rehealed when it is damaged and can be fully recycled at room temperature and therefore provides a reliable, economical, and ecofriendly solution to wearable technologies. In the following, we will present our work on device design, mechanical properties, rehealing and recycling of the strain sensor, characterization of strain sensing, and application of the strain sensor on joint motion and respiration monitoring.

## 2. Results

### 2.1. Device Design and Demonstration

**Figure 1:**
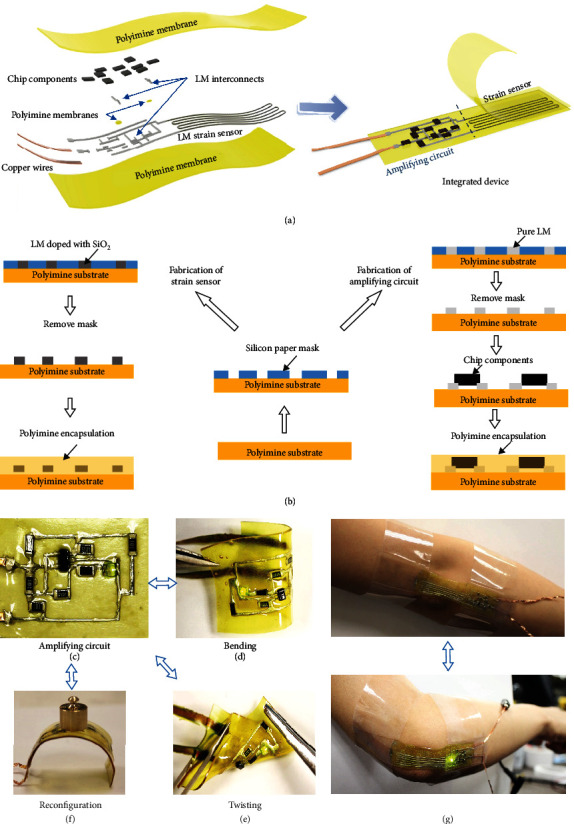
Design, construction, and fabrication of the stretchable strain sensing system. (a) Exploded view of the strain sensing system. (b) Schematic illustration of the fabrication processes of the amplifying circuit and strain sensor. Optical images of the amplifying circuit in its undeformed (c), bent (d), and twisted (e) states. (f) Reconfiguration of the amplifying circuit. (g) The strain sensing system is attached on an elbow for monitoring its flexion states. When the elbow is straight, the LED is off (top). When the elbow flexes beyond a threshold, the LED turns on (bottom).

An exploded view of the stretchable strain sensing system is shown in Figure 1(a) to illustrate the design and construction of the device. It consists of two subsystems: the amplifying circuit and the strain sensor. The amplifying circuit incorporates eutectic gallium-indium liquid metal (LM) alloy as intrinsically stretchable and self-healable circuit interconnects between commercial small-scale chip components. The strain sensor is made of gallium-indium liquid metal (LM) alloy doped with 6%wt SiO_2_ microparticles (40 *μ*m diameter, Sigma-Aldrich), in order to enhance strain sensitivity. As a result, the SiO_2_-doped LM has a resistivity of 95 × 10^−6^ *Ω*·cm (conductivity 1.05 × 10^6^ S/m), and the GF of the strain sensor can be increased to 2.5, which is 2.58 times of the pure EGaIn strain sensors [28]. Furthermore, adding 6%wt SiO_2_ microparticles to LM increases the viscosity while maintaining the fluidity. This improves the printability, so that the mixed LM can be easily screen printed onto the polyimine film against a mask at room temperature [60]. Both subsystems are encapsulated by hyperelastic polyimine membranes (Young's modulus *E* = 2 MPa), which can be synthesized from commercially available monomers (Figure S1). Two copper wires are used to connect the device to an external power supply. The detailed fabrication process is illustrated in Figure 1(b). The fluidity of LM circuitry renders the device superior compliance and stretchability without sacrificing its excellent electronic performance. Compared with serpentine interconnects widely used in stretchable electronics [14, 53–55], the design and manufacturing of the device reported herein are much simpler and more economical. The soft integrated device (Figure 1(c)) can be bent (Figure 1(d)) and twisted (Figure 1(e)) while functioning. Because bond exchange reactions within the polyimine network can effectively relax residual stresses, the device has excellent malleability. It can be reconfigured into different shapes for different purposes. For example, Figure 1(f) shows that the device is bent into a semicircle and kept at the new shape after stress relaxation by heating the device to 60°C. When necessary, the device can be reconfigured into other shapes, and this process is reversible. Combining the stretchable strain sensor with an amplifying circuit composed of semiconductor micro/nanochips can realize a fully functional wearable system, which can provide superior electronic performance (see Supplementary Materials for details (available here)). For example, the integrated device can be mounted around a joint, for real-time monitoring of the joint motion. As shown in Figure 1(g), the device is attached on an elbow (top); when the bending of the elbow exceeds a predetermined threshold, the LED turns on (bottom) to warn the wearer.

### 2.2. Mechanical Properties

**Figure 2:**
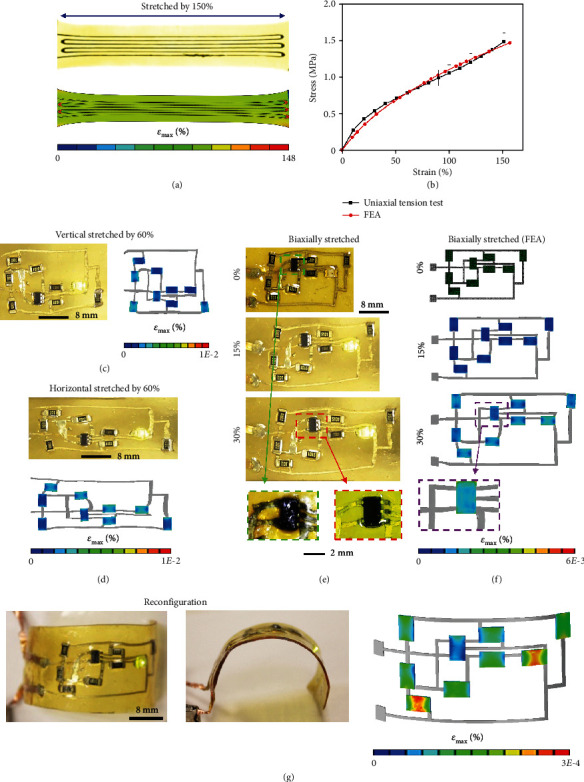
Mechanical properties of the strain sensor and amplifying circuit. (a) Optical image (top) and FEA strain contour (bottom) of the strain sensor stretched by 150%. (b) Uniaxial stress-strain curves of the strain sensor obtained from experiment and FEA. (c) Optical image (left) and FEA strain contour (right) of the amplifying circuit vertically stretched by 60%. (d) Optical image (top) and FEA strain contour (bottom) of the amplifying circuit horizontally stretched by 60%. (e) Optical images of the amplifying circuit at 0% strain (top), biaxially stretched by 15% (middle), and biaxially stretched by 30% (bottom). The two insets show microscope images of a chip component at undeformed and biaxially stretched by 30% states. (f) FEA simulation model of the amplifying circuit (top), and strain contours of the amplifying circuit biaxially stretched by 15% (middle) and 30% (bottom). (g) The amplifying circuit can be reconfigured to a cylindrical shape (left: top view, middle: side view). FEA simulation gives the strain contour in chip components in the cylindrical shape (right).

The image of a LM strain sensor stretched by 150% is shown in Figure 2(a) (top), and the stress-strain curve is presented in Figure 2(b). The effective modulus of the LM strain sensor is obtained to be 2 MPa. Since the chip components are rigid and fragile, finite element analysis (FEA) can provide strain distribution in these components when the strain sensor is subjected to large deformation, in order to verify that they are safe under such conditions. Finite element analysis (FEA) of the LM strain sensor under uniaxial tension was performed. The strain contour of the LM strain sensor being stretched by 150% is shown in Figure 2(a) (bottom). The stress-strain curve obtained from FEA is given in Figure 2(b), which shows good agreement with experimental results.

The mechanical performance of the stretchable amplifying circuit was investigated, and the results are presented in Figures 2(c)–2(g). Figures 2(c) and 2(d) show the amplifying circuit being stretched by 60% along vertical and horizontal directions, respectively. The strain contours of the chips shown in Figures 2(c) and 2(d) demonstrate that the maximum strains in the chips are smaller than 0.01% even though the strains in polyimine reach ~140% (Figures S2(a) and S2(b)). The maximum strains in chips are much lower than the typical failure strain of silicon (~1%), implying safe operation of the amplifying circuit under such extreme deformations.  Figure 2(e) presents images of the amplifying circuit when no strain (top), 15% biaxial strain (middle), and 30% biaxial strain (bottom) are applied. The enlarged microscope images of a chip component before and after application of 30% biaxial strain, as shown at the bottom of Figure 2(e), show no signs of debonding or failure in the LM interconnects at the edges of the chip component. The excellent connection between interconnects and chips is attributed to the good wettability between LM and metal pins [26, 56, 61, 62]. Enlarged microscope images of a chip component under uniaxial tension up to 60% strain are shown in Figure S3. These results clearly manifest the robustness of the LM circuitry under extreme deformations. FEA simulation results of the amplifying circuit under biaxial stretching are presented in Figure 2(f), which show good agreement with the corresponding experimental results in Figure 2(e). The maximum strains in the chip components are lower than 0.006% even when the polyimine substrate experiences ~100% strain (Figure S2(c)).

Thanks to the bond exchange reactions within the polyimine network [56–58], the integrated device can be reconfigured into different shapes. An originally flat device was reconfigured into a cylindrical shape in Figure 2(g). This reconfiguration is because the stress built inside the polyimine network during bending can be effectively relaxed due to bond exchange reactions at 60°C. This process is reversible and can be repeated multiple times to achieve different shapes. Such capability renders the integrated sensing device excellent conformability to complex surfaces without introducing excessive interfacial stresses, which is beneficial for long-term reliability. Strain contours of the chip components and polyimine obtained from FEA simulation are shown in the right frame of Figures 2(g) and S2(d), respectively.

### 2.3. Rehealing and Recycling

**Figure 3:**
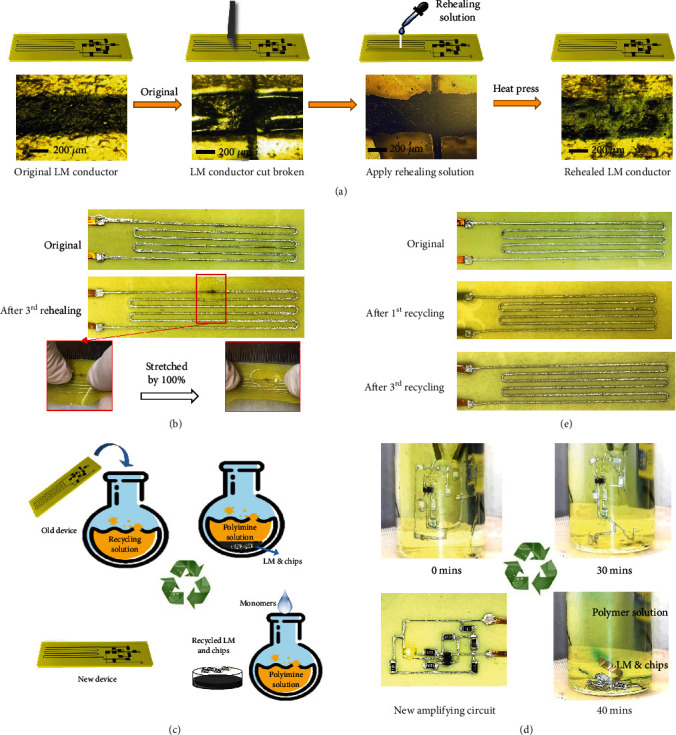
Rehealing and recycling of the device. (a) Schematic illustration (top) and experimental microscope images (bottom) of the rehealing process. (b) Optical images of the original strain sensor (top) and after cutting and rehealing three times (middle). The rehealed strain sensor can be stretched by 100% (bottom). (c) Schematic illustration of the recycling process. (d) Optical images of the recycling of an amplifying circuit. (e) Optical images of the original strain sensor (top) and after recycling for once (middle) and three times (bottom).

Because of bond exchange reactions within the polyimine network and flowability of LM, the integrated strain sensor has excellent rehealability when it is damaged.  Figure 3(a) illustrates the detailed rehealing process of a LM conductor encapsulated by polyimine, with schematics shown at the top and experimental optical microscope images shown at the bottom (see Materials and Methods for details). The damaged interface (Figure 3(a), second frame) of the strain sensor can be rehealed, and no sign of crack can be seen even under a microscope (Figure 3(a), fourth frame). It is worth pointing out that the rehealing process in polyimine generates new oligomers/polymers across the broken interfaces and leads to covalent (chemical) bonding of the two pieces of polyimine. This process mimics the healing of human skin, and no interfaces exist at the cut area after rehealing. This mechanism is intrinsically different from bonding two parts together using glue, which generates physical bonding (van der Waals interactions) at the interface and usually leads to significant degradation in mechanical properties. Furthermore, the strain sensor can reheal multiple times. As shown in Figures 3(b) and S6, the strain sensor can be still stretched by 100% after three-time rehealing.

When the device is seriously damaged or no longer needed, the whole integrated sensor can be completely recycled without leaving any waste behind. The recycling process of an amplifying circuit is schematically illustrated in Figures 3(c) and 3(d) shows optical images of the experimental recycling process. It starts with immersing the old device in the recycling solution. Excessive free primary amines in the recycling solution can react with the imine-linked network through transimination, which leads to depolymerization of polyimine into oligomers/monomers that are soluble in the solvent, and LM and chip components sink to the bottom (top right, Figures 3(c) and 3(d), and bottom right, Figure 3(d)). Both LM and chip components can be easily separated from the solution (bottom right, Figure 3(c)). Then, terephthalaldehyde can be proportionally added into the recycled solution for the synthesis of new polyimine. The recycled polyimine, LM, and chip components can be reused for making a new device (bottom left, Figures 3(c) and 3(d)). The recycling of a strain sensor is demonstrated in Figure S4 and Supplementary Movie S1. The recycling processes can be completed within 40 mins and 18 mins at room temperature for the amplifying circuit and strain sensor, respectively. The strain sensor can also recycle multiple times. Figures 3(e) and S7(a) show the device after 1^st^ recycling, 2^nd^ recycling, and 3^rd^ recycling.

### 2.4. Strain Sensor Characterization

**Figure 4:**
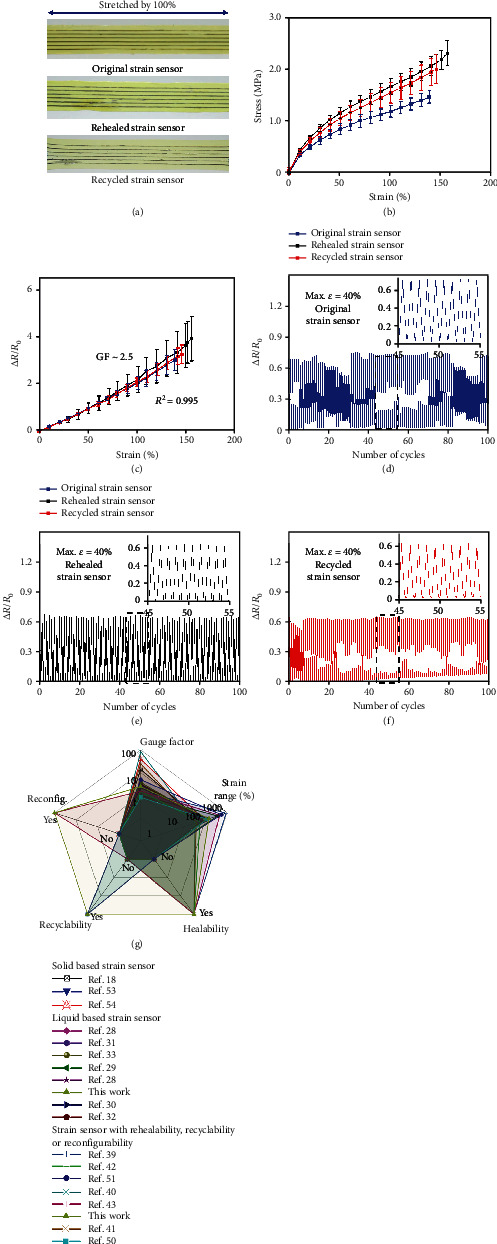
Characterization of the rehealed and recycled strain sensor. (a) Optical images of the original (top), rehealed (middle), and recycled (bottom) strain sensor stretched by 100%. (b) Stress-strain curves of the original, rehealed, and recycled strain sensors. (c) Relative resistance change Δ*R*/*R*_0_ of the original, rehealed, and recycled strain sensors versus applied uniaxial strain. Relative resistance change Δ*R*/*R*_0_ of the original (d), rehealed (e), and recycled (f) strain sensor under cyclic loading. The maximum strain is 40%. The insets exhibit magnified views of 10 cycles between cycle numbers 45 and 55. (g) Comparison of this work with previously reported stretchable strain sensors.

Figure 4 compares the mechanical and electrical properties of the original, rehealed, and recycled strain sensors. As shown in Figure 4(a), the rehealed and recycled strain sensors can be stretched by 100%, without mechanical failures (Supplementary Movie S2). Stress-strain curves of the original, rehealed, and recycled strain sensors are presented in Figure 4(b) and show comparable mechanical behavior under uniaxial stretching. As a resistance-based strain sensing device, the LM strain sensor can be characterized using four-point resistance measurement. Two copper wires were used to connect the two ends of the LM strain sensor with the measurement setup (inset of Figure S5). The measured resistance versus applied uniaxial strain is presented in Figure S5. Figure 4(c) compares the relative resistance change Δ*R*/*R*_0_ of the original, rehealed, and recycled strain sensors due to uniaxial strain, and no noticeable differences can be observed. It is worth pointing out that the LM strain sensor can be rehealed and recycled multiple times. As shown in Figure S6(a), the strain sensor can still be stretched by 100% after three-time rehealing. The stress-strain curves of the rehealed devices show elasticity comparable to the original device after three-time rehealing (Figure S6(b)). The slightly increased stiffness of the rehealed devices is probably due to the heat pressing process during rehealing. Figure S6(c) shows that the relative resistance change versus applied strain of rehealed devices is comparable to that of the original strain sensor. Figure S7(a) illustrates images of an original strain sensor and the device after 1^st^ recycling, 2^nd^ recycling, and 3^rd^ recycling. Figures S7(b) and S7(c) present the stress-strain curves and relative resistance change-strain curves of the original and recycled strain sensors, respectively. They show comparable mechanical and electrical properties, even after three-time recycling. Cyclic tensile tests using 40% strain were performed to assess the reliability of the strain sensor when mounted on human skin (the max strain of human skin is ~40% [63–66]). Figure 4(d) shows the relative resistance change Δ*R*/*R*_0_ of the original strain sensor under cyclic loading, with 40% maximum strain. The inset exhibits a magnified view of 10 cycles between cycle numbers 45 and 55. After 100 cycles, no significant changes can be seen in its response. The results obtained from the rehealed and recycled strain sensors under the same cyclic loading conditions are presented in Figures 4(e) and 4(f). They show comparable performance to that of the original strain sensor.

Figure 4(g) compares our strain sensor with stretchable strain sensors reported in the literature. Five performance indexes are chosen for the comparison, including gauge factor, strain range, rehealability, recyclability, and reconfigurability (refer to Tables S1 and S2 for details). The strain sensor in this work shows gauge factor and strain range comparable to most of the reported stretchable strain sensors. A few previous studies have demonstrated rehealing or recycling, but our device is by far the first strain sensor that can achieve rehealing, recycling, and reconfiguration capabilities simultaneously. When compared with the serpentine strain sensors [53, 54], this work has larger stretchability and better linearity because of the fluidity of LM. In addition, serpentine strain sensors could experience fatigue issues when subjected to cyclic loading, especially at the curved regions where stress concentration occurs, while LM has no stress and fatigue issues. Furthermore, the design and manufacturing of the LM strain sensor are much simpler and cheaper.

### 2.5. Joint Motion and Respiration Monitoring

**Figure 5:**
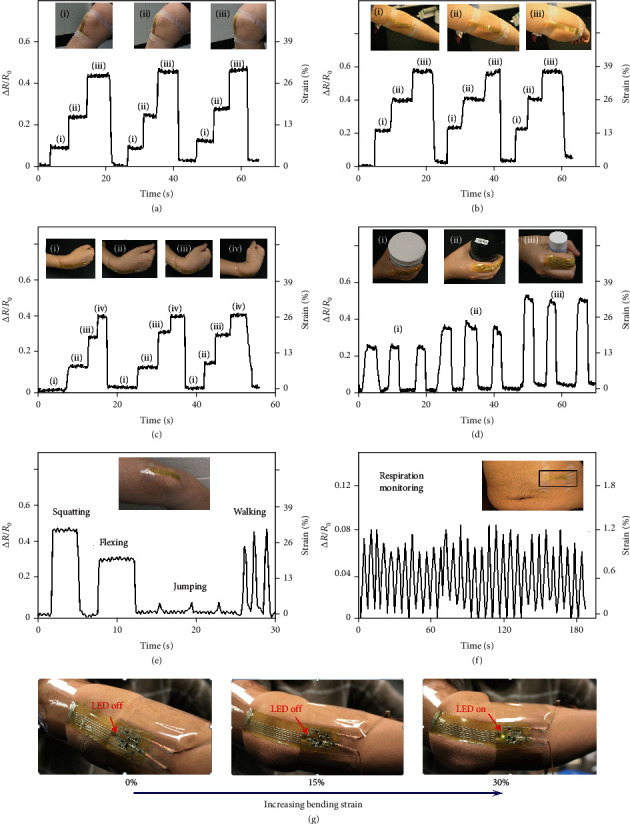
Joint motion and respiration monitoring. The strain sensor is attached on a knee (a), an elbow (b), and a wrist (c) for monitoring the strains induced by joint flexion. (d) The strain sensor is attached on an index finger for sensing the strains induced by holding objects of different sizes. (e) The strain sensor attached on a knee can detect different states of physical activities, including squatting, flexing, jumping, and walking. (f) The strain sensor is attached on the abdomen for monitoring respiration. (g) The integrated strain sensor is attached on an elbow. When the elbow is not flexing (left), or the flexion angle is small (middle), the LED stays off. When the elbow flexion angle is too large (right), the LED turns on.

Strains on human skin can vary between ~1% on the abdomen during breathing to ~40% on joints during flexing, and such information can be useful for health monitoring [63–66]. The compliant and stretchable strain sensor reported here can capture the full range of strains on human skin and thus can be attached onto different parts of human body for different purposes.  Figure 5(a) demonstrates a strain sensor attached on the knee for monitoring its degree of flexion. As shown in insets (i), (ii), and (iii), three different bending states of knee flexion can be accurately captured by the strain sensor, with the maximum tensile strain equal to 30% when the knee is at a 90° angle. The strain sensor can also be attached onto the elbow and waist and can accurately measure different strains at these joints due to different flexing states, as shown in Figures 5(b) and 5(c), respectively.  Figure 5(d) shows a strain sensor attached on an index finger, and the strain information can be used to detect the size of objects held by the hand. The three insets (i), (ii), and (iii) give three objects of diameters 90 mm, 58 mm, and 25 mm, which correspond to the average strains of 17%, 22%, and 33% measured by the strain sensor, respectively. This capability can be applied on robots for accurate sensing of environment and delicate handling of fragile objects [67–69]. When the strain information is combined with frequency data, the strain sensor can provide monitoring of more complex joint motion and physical states.  Figure 5(e) shows four different states of knee joint motion recorded by the strain sensor. The squatting, flexing, jumping, and walking states show significantly different signatures, when strain level, plateau width, and frequency are all taken into consideration. In addition to joint motion, the strain sensor can also be applied on the abdomen for real-time monitoring of respiration.  Figure 5(f) presents respiration monitoring data obtained from the strain sensor, with a frequency of 11/minute. Such information can be used to detect abnormalities in the rate and pattern of respiration, which are a strong indicator of acute events, such as cardiac arrest, chronic obstructive pulmonary disease (COPD), pneumonia, and asthma [70, 71]. Based on the characterization of the strain sensor, the integrated strain sensing system combining strain sensor as a rheostat with a Wheatstone bridge differential amplifier circuit can realize real-time signal processing.  Figure 5(g) shows such an integrated strain sensing system attached on an elbow. When the elbow is not flexing (left), or the flexion angle is small (middle), the strain detected by the strain sensor is small, and the LED stays off. When the elbow flexion angle is too large (right), the strain detected by the strain sensor exceeds the predetermined threshold, leading to the LED to turn on (Supplementary Movie S3). By combining the strain sensor with the semiconductor micro/nanochips, the liquid (the EGaIn alloy), soft material (polyimine film), and hard material (electronic component chips) can be heterogeneously integrated as wearable electronics through a low-cost and quite easy fabrication technology, which can realize the superior electronic performance. This capability of real-time monitoring and warning of the strain at joints can provide assistance during joint rehabilitation [72–75].

## 3. Discussions

To conclude, this study presents an integrated strain sensing system with superior stretchability, rehealability, recyclability, and reconfigurability. These characteristics are realized by integrating dynamic covalent thermoset polyimine as the substrate and encapsulation, eutectic liquid metal alloy as the strain sensing unit and interconnects, and off-the-shelf chip components for measuring and magnifying functions. The rehealed and recycled strain sensor exhibits mechanical and electrical properties comparable to the original device. This strain sensor has been applied to different parts of the body, including the knee, elbow, wrist, finger, and abdomen, for joint motion and respiration monitoring. Such a strain sensing system can find wide applications in health care, prosthetics, robotics, and biomedical devices. Furthermore, rehealability and full recyclability of the integrated device could greatly improve the economics and reduce the environmental impact of electronics.

## 4. Materials and Methods

### 4.1. Polyimine Preparation

The polyimine was synthesized by mixing terephthalaldehyde (0.5 g, 3.72 mmol, Combi-Blocks), 3,3′-diamino-N-methyldipropylamine (0.417 g, 2.87 mmol, Sigma-Aldrich), and tris(2-aminoethyl)amine (0.084 g, 0.574 mmol, Oakwood Chemical) in methanol as shown in Figure S1. The solution was vigorously stirred (Vortex-Genie (G560) SI-0236, Scientific Industries, United States) and poured into a silicone paper mold. Then, evaporating the methanol solvent in the solution in a fume hood for 6 hours at room temperature and heat-pressing at 80°C and 8.5 kPa for 12 hours led to cured polyimine films.

### 4.2. LM Preparation

The pure LM used as interconnects was eutectic metal alloy consisting of gallium (75%) and indium (25%) (EGaIn, Sigma-Aldrich), which maintains a liquid state at room temperature (melting point 15.7°C) and has a resistivity of 29.4 × 10^−6^ *Ω*·cm and conductivity of 3.4 × 10^6^ S/m [28, 76]. In order to increase the resistivity of LM for improved strain sensitivity, the EGaIn was mixed with 6%wt SiO_2_ microparticles (40 *μ*m diameter, Sigma-Aldrich) and stirred in air at 500 rpm for 2 mins and at 2000 rpm (vigorously stirred) for 8 mins.

### 4.3. Fabrication and Characterization of a Stain Monitoring Device

As shown in Figure 1(b), a silicon paper mask, made by laser cutting (LIDE laser cutting machine) a 0.2 mm thick silicone paper film (Ruspepa nonstick silicone paper), was laminated over a weakly adhering polyimine membrane substrate, and pure and SiO_2_-doped LM were dispensed over the circuit and sensor mask, respectively. Then, a razor blade was used to remove excessive materials. By putting the membrane on a cold plate (DC10 microprocessor controlled dry bath incubator, Hangzhou Ruicheng Instrument, China) at -10°C for 30 min, the LM solidified (EGaIn solidifies at temperature below 15.7°C). The silicon paper mask was then peeled off, and the solidified LM traces were left on the polyimine membrane. The commercial chips' pins were placed onto the designated LM contact pads manually, with the placement accuracy assured using an optical microscope, similar to PCBs but without the need of soldering. It is worth pointing out that the chips were well bonded to LM without using conductive paste or solder, because the LM had good wettability with metal pins, which led to a robust connection between LM and chip components even under large deformation. Pouring the same formula polyimine solution over the entire device encapsulated the liquid metal and chips. After curing at room temperature, the integrated device was obtained. At intersection, insulating polyimine was used to separate two LM interconnects (Figure S8). Moreover, two copper wires were used to connect the device to an external power source.

### 4.4. Rehealing, Recycling, and Reconfiguring Process

To reheal polyimine films, an original LM conductor (Figure 3(a), first frame) was cut broken by a razor blade, and the crack had a width of ~80 *μ*m, as shown by the microscope image (Figure 3(a), second frame). A small drop of rehealing agent (the same formula as polyimine solution) was added to the crack (Figure 3(a), third frame), followed by heat-pressing at 80°C and 8.5 kPa for 10 mins. The interface was then rehealed, and no sign of crack can be seen even under a microscope (Figure 3(a), fourth frame). To recycle polyimine films, 3,3′-diamino-N-methyldipropylamine (0.417 g, 2.87 mmol) and tris(2-aminoethyl)amine (0.084 g, 0.574 mmol) were mixed in methanol. Such recycling solution depolymerizes the polyimine network into oligomers/monomers which can dissolve in methanol. After separating the LM and chip components from the polymer solution, terephthalaldehyde (0.5 g, 3.72 mmol) was added into the polymer solution for regenerating polyimine membrane. Dilute hydrochloric acid can remove the oxide compounds on the surface of the LM and reunite them [77]; then, both LM and chips can be cleaned by methanol for reuse (Supplementary Movie S1). To reconfigure the device, the originally flat device was mounted onto a cylinder of diameter 27.5 mm at 60°C. After cooling down at room temperature for 10 minutes, the cylindrical shape was retained (Figure 2(g)).

### 4.5. Tensile and Cyclic Mechanical Test

Polyimine and LM strain sensor were tested using an INSTRON mechanical testing system (INSTRON 5965 with 50 N load cell, Instron, Norwood, MA). A loading strain rate of 0.003/s was used for a quasistatic tensile test until the polyimine film broke and for a 40% strain cyclic test for 100 cycles [78, 79]. Four-point measurement was adopted to measure the resistance change of the strain sensor. A current supplier (HY3005M-3 Digital Control) was used for the current input, and Arduino as well as 16-bit analog to digital convertor (ADS1115) was used for measuring the voltage every 0.1 seconds. A constant current of 10 mA was applied on the strain sensors.

### 4.6. Wheatstone Bridge and Differential Amplifying Circuit

In the integrated device, the resistances of LM interconnect and strain sensor were ~0.5 *Ω* and ~5.4 *Ω*, respectively. The on/off states for the LED are controlled by a bridge amplifier circuit composed of several resistors and an operational amplifier. The monitoring circuit consisted of a Wheatstone bridge and a stage of differential amplification. The resistor values for three fixed arms of the Wheatstone bridge (*R*_01_ = 100 *Ω*, *R*_11_ = 100 *Ω*, and *R*_2_) were determined by circuit simulation (Figure S9). As shown in Figure S10, the resistance of *R*_2_ was selected in order to illuminate the LED at selected threshold of strain (for example, 7.4 *Ω* resistor was selected in order to illuminate the LED at ~20% strain). As a result, the sensing system can detect the strain and give a warning in real time (Supplementary Video S3), which can be applied to many parts of the body as shown in Figure 5.

The resistors of the monitoring circuit (Figure S9) were type 1206 thick film resistors (3.2 mm × 1.6 mm × 1.0 mm, ERJ series, Panasonic Electronic Components, USA). The amplification of the voltage offset was done by using a 5-Lead SOT-23 amplifier chip (2.9 mm × 2.8 mm × 1.45 mm, AD8505, Analog Devices, USA). The indicator LED (3.2 mm × 1.6 mm × 1.1 mm, LTST-C230KGKT, Lite-On Inc., USA) was a surface mount chip component with dimensions similar to 1206 components. The resistance values in the Wheatstone bridge and output voltage of amplifier circuit were determined by a circuit simulation package (LTspice, Linear Technology Corporation, USA). The final device configuration of the respiration sensor was completed by connecting a power source or a thin lithium polymer battery (3.7 V, 45 mAh, GMB, China) to the power terminals.

### 4.7. FEA Simulation

Finite element analysis (FEA) was conducted using a commercial software package ABAQUS. For simplicity, the LM circuitry was modeled as an extremely soft incompressible material with Young's modulus 3 orders of magnitude smaller than polyimine, which does not noticeably affect the mechanical behavior of the wearable electronic system. Young's moduli of the chip components, polyimine, and LM were 160 GPa, 2 MPa, and 3 kPa, respectively. Poisson's ratios were 0.45, 0.35, and 0.5, respectively. The polyimine and LM interconnects were modeled as Neo-Hookean hyperelastic materials using 3D hybrid stress elements (C3D8H), and the chips were modeled as elastic material using 3D stress elements (C3D8).

A Neo-Hookean material model was used to take into account the nonlinear stress-strain relation of the hyperelastic material; the material coefficients *C*_10_ and *D*_1_ are written as

(1) $$\begin{array}{c} C_{10}=\frac{E}{4\left(1+v\right)},\\ D_{1}=\frac{6\left(1-2v\right)}{E}. \end{array}$$

Thus, the material coefficients in Neo-Hookean models in ABAQUS were *C*_10_ = 0.3704 and *D*_1_ = 0.9000 for polyimine and *C*_10_ = 0.0005 and *D*_1_ = 0 for LM.

### 4.8. Sensing Performance Determination

The performances of strain sensors, including gauge factor (GF), linearity, and strain range, are listed in Tables S1 and S2. The GF can be calculated as

(2) $$\begin{array}{c} \text{GF}=\frac{∆R/R_{0}}{\varepsilon }, \end{array}$$

in which *R*_0_ is the resistance of a strain sensor before deformation, Δ*R* is the resistance change due to deformation, and *ε* is the applied strain.

The linearity of a strain sensor can be obtained by calculating the linear regression coefficient of determination (*R*^2^) from Δ*R*/*R*_0_ versus *ε* curve in Figure 4(c). The closer the coefficient of determination (*R*^2^) to 1, the stronger the linear relationship. Our strain sensor gives *R*^2^ = 0.995.

The strain range is another important indicator. A stretchable strain sensor should keep conductive when subjected to mechanical stretch. The strain range is obtained when the failure of the strain sensor happens. The failure could occur either in the conductive elements or in the substrate. In this work, the maximal strain was obtained when the substrate (or LM trace) failed, which gave the strain range to be 0~160%.

## Data Availability

All data are available in the main text or the supplementary materials.

## Supplementary Materials

Supplementary Materials Supplementary 1: the mechanism of Wheatstone bridge and differential amplifying circuit. Figure S1: preparation of the polyimine network through imine condensation reaction. Figure S2: max principal strain contours in polyimine when the amplifying circuit is subjected to 60% uniaxial strain along vertical (a) and horizontal (b) directions, 30% biaxial strain (c), and reconfiguration (d). Figure S3: optical images of the amplifying circuit under uniaxial stretching. The second image shows the device after release of the strain, to illustrate reversibility in the responses. Microscope images of op-amplifier at the bottom show no signs of debonding or failure in LM interconnects. Figure S4: optical images of recycling process of the strain sensor. Figure S5: resistance change of the strain sensors versus applied uniaxial strain. Figure S6: (a) optical images of the original strain sensor (top) and after cutting and rehealing for once (second row), twice (third row), and three times (bottom). The rehealed strain sensor can be stretched by 100%. (b) Stress-strain curves of the original and rehealed strain sensors. (c) Relative resistance change Δ*R*/*R*_0_ of the original and rehealed strain sensors versus applied uniaxial strain. Figure S7: (a) optical images of the original strain sensor (top left) and after recycling for once (top right), twice (bottom left), and three times (bottom right). (b) Stress-strain curves of the original and recycled strain sensors. (c) Relative resistance change Δ*R*/*R*_0_ of the original and recycled strain sensors versus applied uniaxial strain. Figure S8: the amplifying circuit without chip components (left). Enlarged microscope image shows details of intersections of LM wires (right). Figure S9: Wheatstone bridge differential amplifier circuit. Figure S10: simulated *V*_out_ versus *R*_sensor_ and the strain *ε* applied to the strain sensor. Table S1: performance summary of recently reported solid- and liquid-based stretchable strain sensors. Table S2: performance summary of recently reported stretchable strain sensors with rehealability, recyclability, or reconfigurability. Supplementary 2: Supplementary Movie S1: recycling process of the strain sensor, and the recycled strain sensor shows robust mechanical properties. Supplementary 3: Supplementary Movie S2: the rehealed and recycled strain sensors subjected to stretching. Supplementary 4: Supplementary Movie S3: the integrated strain sensor attached on an elbow for real-time sensing and warning of joint motion.
