# Supplementary material for: Stretchable, Rehealable, Recyclable, and Reconfigurable Integrated Strain Sensor for Joint Motion and Respiration Monitoring
Source: Research (Wash D C). 2021 Jul 29;2021:9846036. doi: 10.34133/2021/9846036 (PMC8347367; doi:10.34133/2021/9846036)
Supplement: Supplementary Materials — Supplementary 1: the mechanism of Wheatstone bridge and differential amplifying circuit. Figure S1: preparation of the polyimine network through imine condensation reaction. Figure S2: max principal strain contours in polyimine when the amplifying circuit is subjected to 60% uniaxial strain along vertical (a) and horizontal (b) directions, 30% biaxial strain (c), and reconfiguration (d). Figure S3: optical images of the amplifying circuit under uniaxial stretching. The second image shows the device after release of the strain, to illustrate reversibility in the responses. Microscope images of op-amplifier at the bottom show no signs of debonding or failure in LM interconnects. Figure S4: optical images of recycling process of the strain sensor. Figure S5: resistance change of the strain sensors versus applied uniaxial strain. Figure S6: (a) optical images of the original strain sensor (top) and after cutting and rehealing for once (second row), twice (third row), and three times (bottom). The rehealed strain sensor can be stretched by 100%. (b) Stress-strain curves of the original and rehealed strain sensors. (c) Relative resistance change ΔR/R0 of the original and rehealed strain sensors versus applied uniaxial strain. Figure S7: (a) optical images of the original strain sensor (top left) and after recycling for once (top right), twice (bottom left), and three times (bottom right). (b) Stress-strain curves of the original and recycled strain sensors. (c) Relative resistance change ΔR/R0 of the original and recycled strain sensors versus applied uniaxial strain. Figure S8: the amplifying circuit without chip components (left). Enlarged microscope image shows details of intersections of LM wires (right). Figure S9: Wheatstone bridge differential amplifier circuit. Figure S10: simulated Vout versus Rsensor and the strain ε applied to the strain sensor. Table S1: performance summary of recently reported solid- and liquid-based stretchable strain sensors. Table S2: perf [file 9846036.f1.zip › Shi_Revised Supplementary Materials_3nd revision.docx]

Supplementary Materials

Stretchable, Rehealable, Recyclable and Reconfigurable Integrated Strain Sensor for Joint Motion and Respiration Monitoring

Chuanqian Shi^1,2^†, Zhanan Zou^1^†, Zepeng Lei^3^, Pengcheng Zhu^1,4^, Guohua Nie^2^*, Wei Zhang^3^*, and Jianliang Xiao^1^*

*^1^Department of Mechanical Engineering, University of Colorado Boulder, Boulder, Colorado 80309, USA*

*^2^School of Aerospace Engineering and Applied Mechanics, Tongji University, Shanghai 200092, China*

*^3^Department of Chemistry, University of Colorado Boulder, Boulder, Colorado 80309, USA*

*^4^School of Materials Science and Engineering, Beihang University, Beijing, 100191, China*

†These authors contributed equally.

*Correspondence should be addressed to Jianliang Xiao; [Jianliang.Xiao@colorado.edu](mailto:Jianliang.Xiao@colorado.edu), Wei Zhang; [Wei.Zhang@colorado.edu](mailto:Wei.Zhang@colorado.edu) and Guohua Nie; [ghnie@tongji.edu.cn](mailto:ghnie@tongji.edu.cn)

**The mechanism of Wheatstone bridge and differential amplifying circuit:**

Figure S11 illustrates the circuit of the integrated device, which is a Wheatstone bridge differential amplifier circuit composed of several resistors and an operational amplifier. The Wheatstone bridge circuit has four arms, among which three are fixed resistors (R_01_=100Ω, R_11_=100Ω and R_2_) and one is a strain sensor (R_sensor_). Here, the differential amplifier is used as a differential voltage comparator by comparing the input voltage V_1_ with V_2_. Such circuit acts as a switch which switches the LED “on” or “off”, depending on the resistance of the strain sensor R_sensor_. When the strain sensor is not stretched or the applied strain is small, the resistance of strain sensor R_sensor_ is smaller than R_2_, leading to V_1_>V_2_ and the output voltage V_out_=0, the LED stays off. When the strain sensor is stretched beyond a threshold, R_sensor_ is larger than R_2_, leading to V_2_>V_1_ and the output voltage V_out_~3.7V, the LED turns on.

As shown in Figure S12, the circuit simulation results demonstrate the output voltage of the amplifier V_out_ versus the resistance of the strain sensor R_sensor_ and the corresponding strain ε applied to the strain sensor, for different prescribed resistance values of R_2_. The value of R_2_ could be selected as the threshold of the strain sensor to control when the LED switches on. For example, when R_2_=7.4 Ω is selected, the LED switches on when the strain sensor is stretched by 20%. As a result, the integrated strain sensing system can detect the strain and give a real-time warning by switching on the LED (Supplementary Video S3).


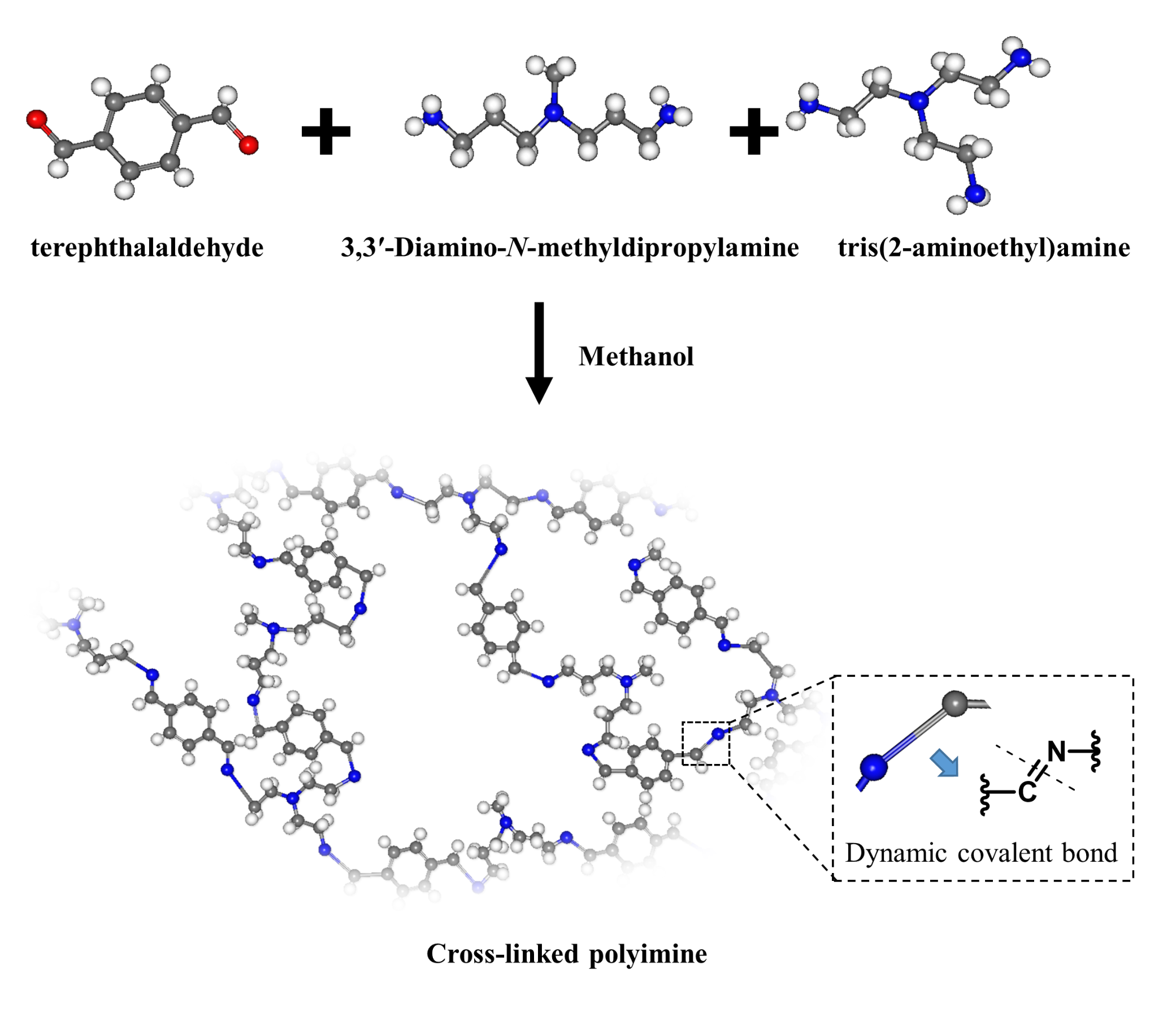


**Figure S1:** Preparation of polyimine network through imine condensation reaction.


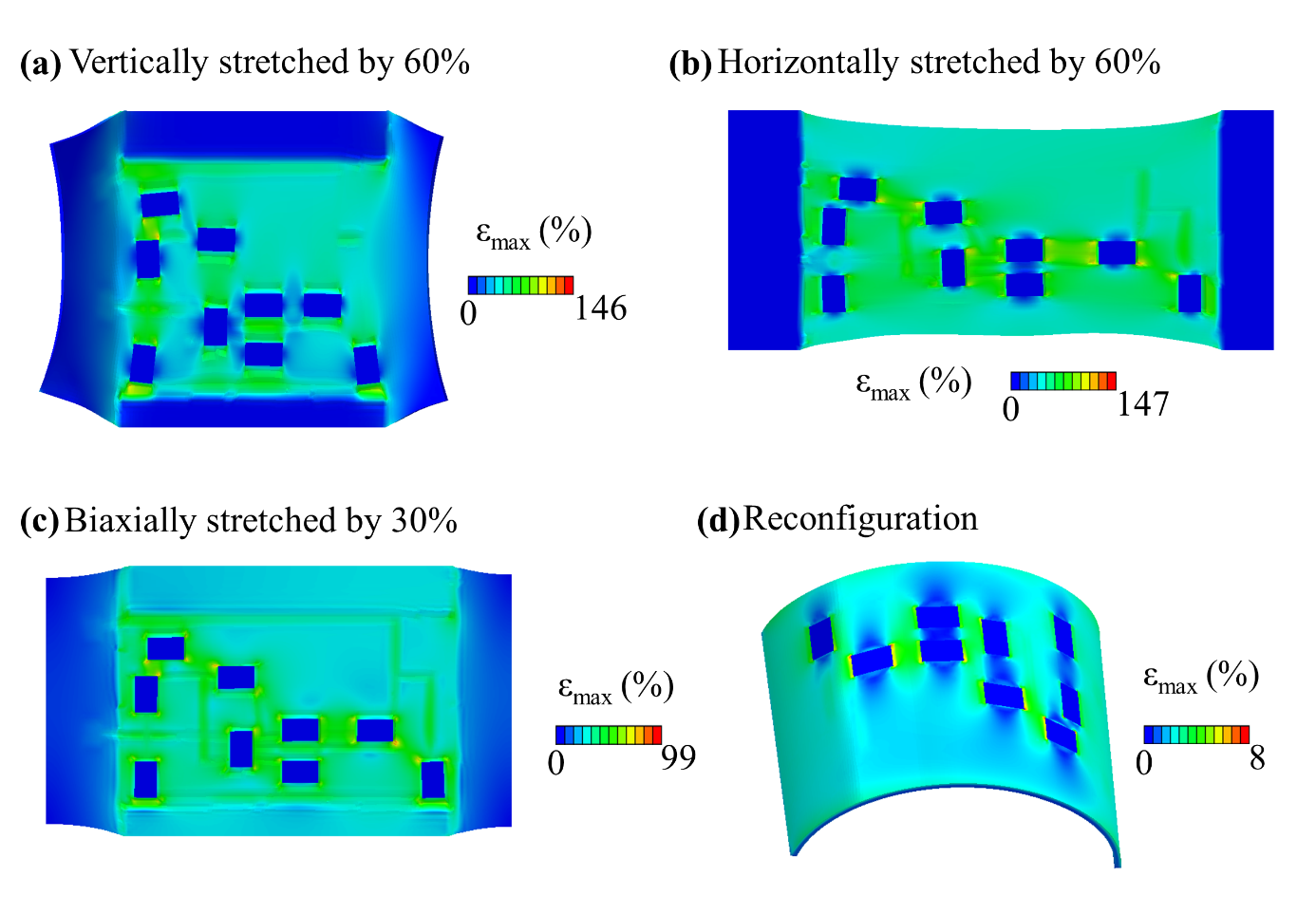


**Figure S2:** Max principal strain contours in polyimine when the amplifying circuit is subjected to 60% uniaxial strain along vertical (a) and horizontal (b) directions, 30% biaxial strain (c), and reconfiguration (d).


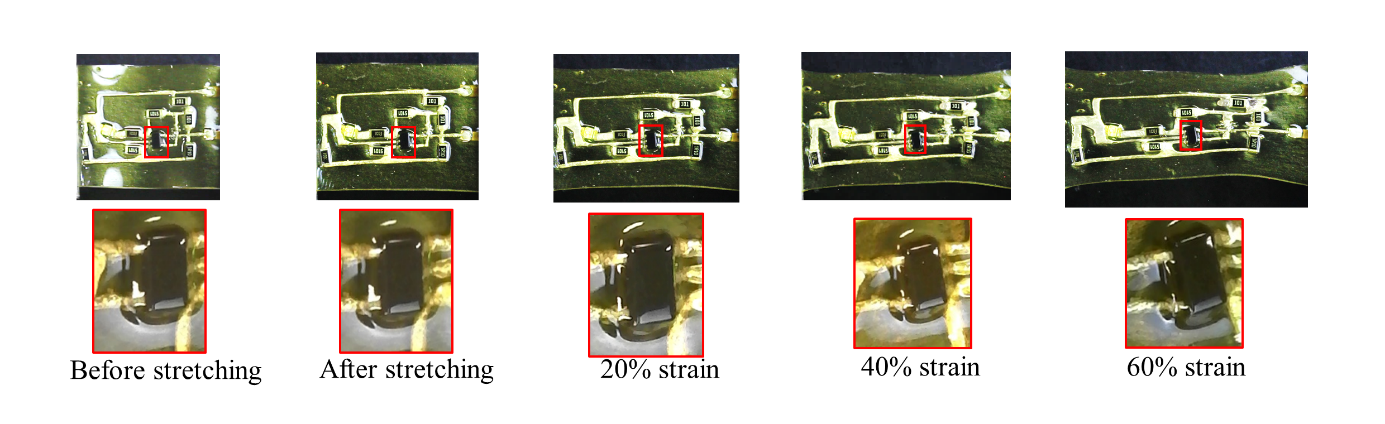


**Figure S3: Optical images of the amplifying circuit under uniaxial stretching.** The second image shows the device after release of the strain, to illustrate reversibility in the responses. Microscope images of op-amplifier at the bottom show no signs of debonding or failure in LM interconnects.


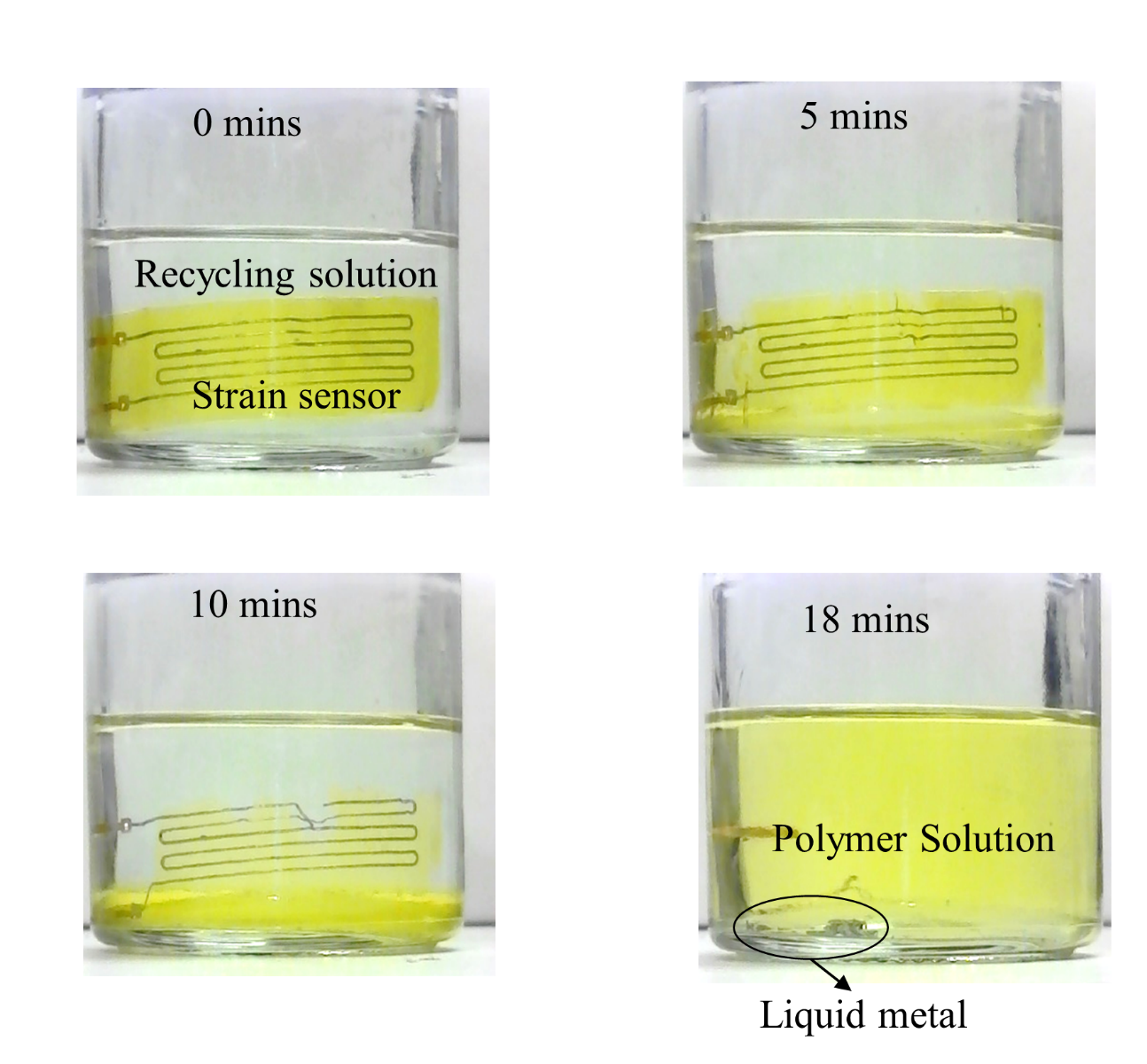


**Figure S4:** Optical images of recycling process of the strain sensor.


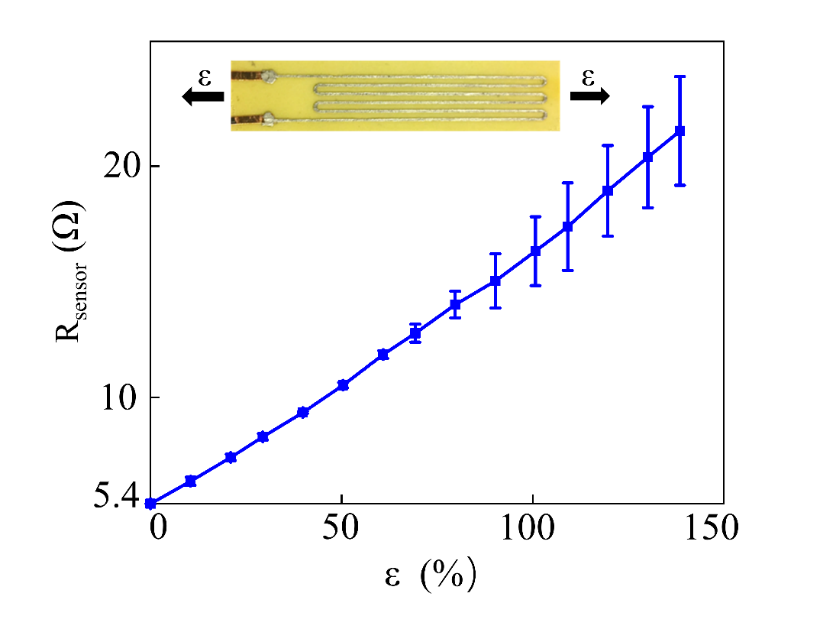


**Figure S5:** Resistance change of the strain sensors versus applied uniaxial strain.

**
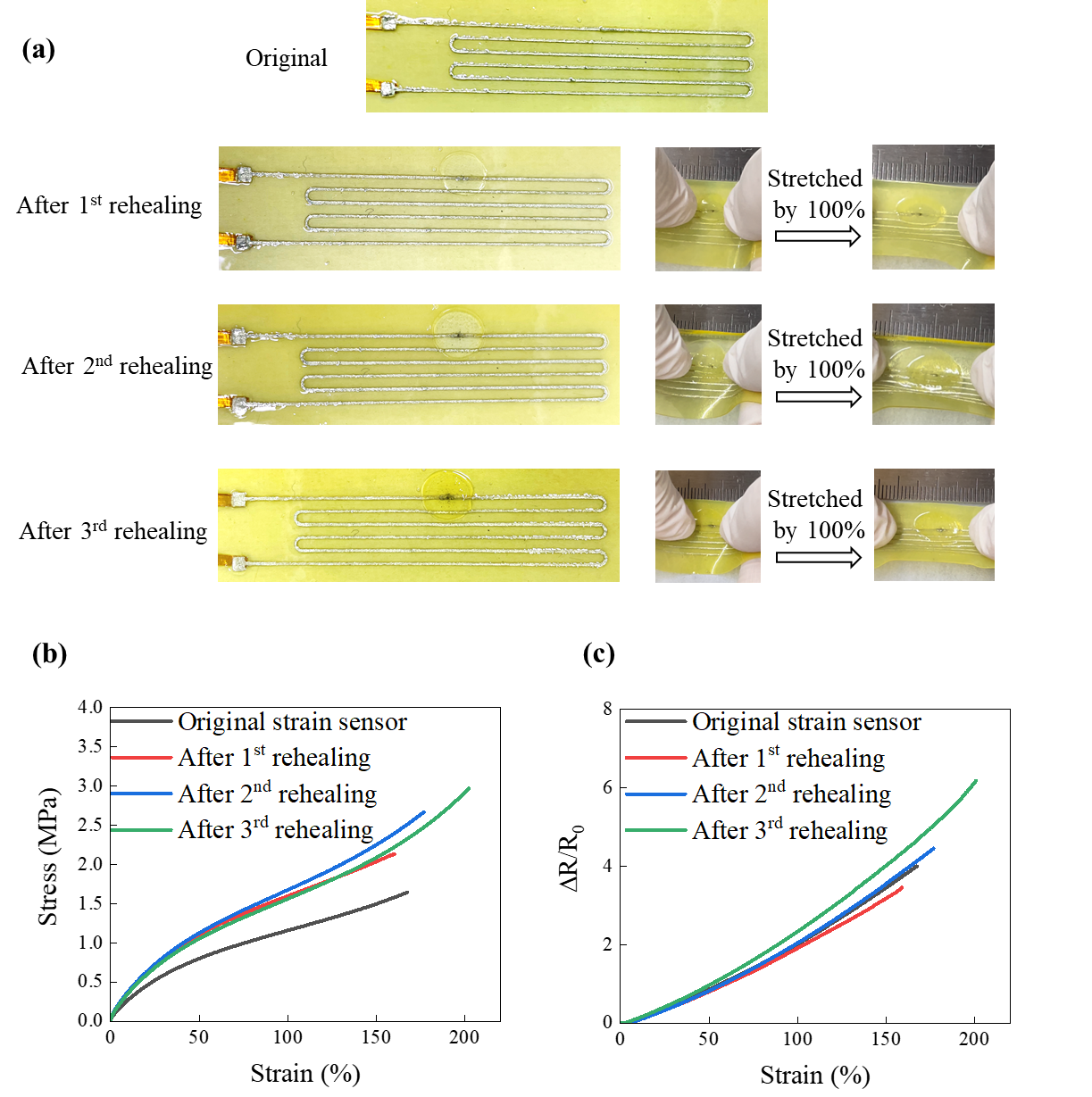
**

**Fig. S6.** (a) Optical images of the original strain sensor (top) and after cutting and rehealing for once (second row), twice (third row) and three times (bottom). The rehealed strain sensor can be stretched by 100%. (b) Stress-strain curves of the original and rehealed strain sensors. (c) Relative resistance change ΔR/R_0_ of the original and rehealed strain sensors versus applied uniaxial strain.


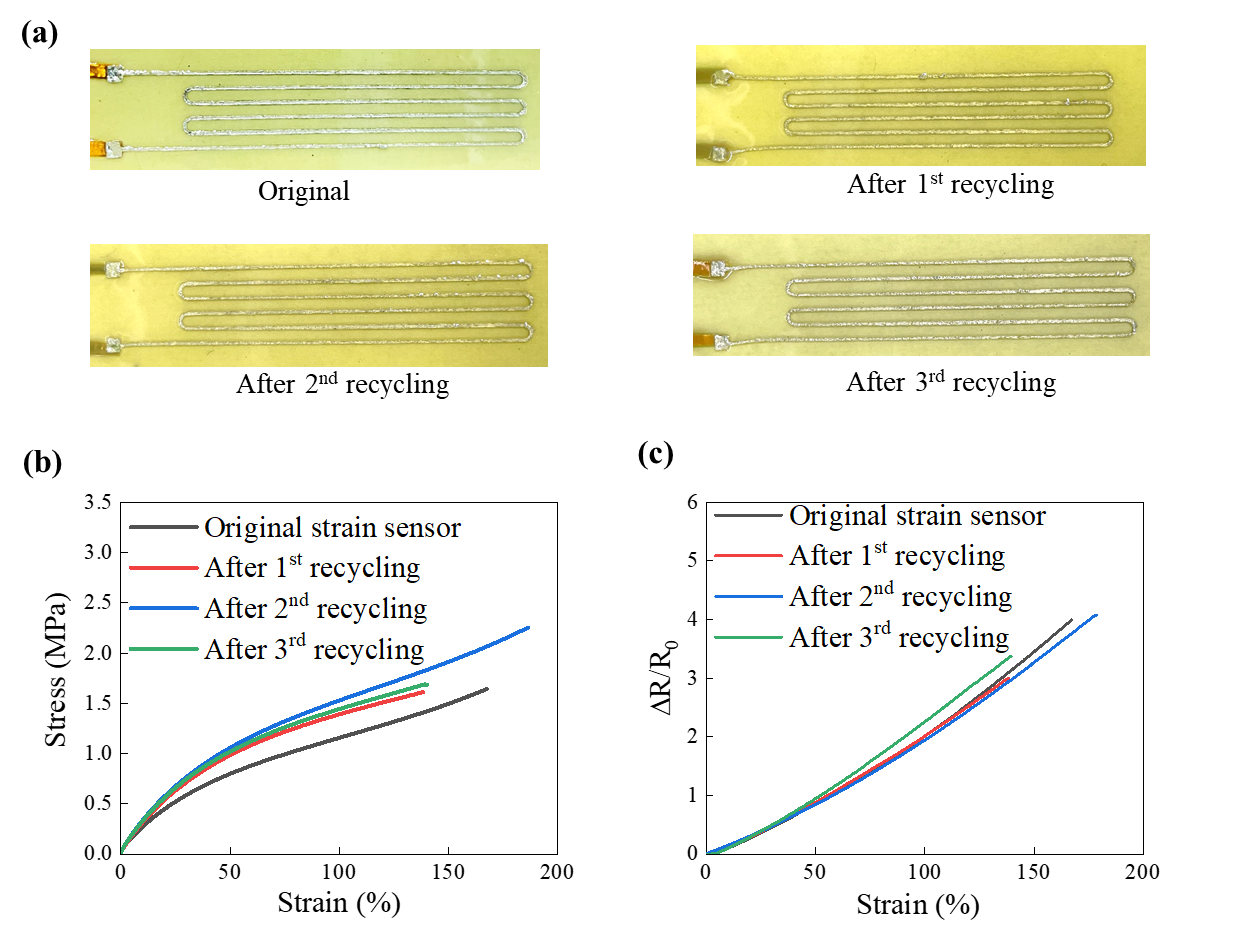


**Fig. S7.** (a) Optical images of the original strain sensor (top left) and after recycling for once (top right), twice (bottom left) and three times (bottom right). (b) Stress-strain curves of the original and recycled strain sensors. (c) Relative resistance change ΔR/R_0_ of the original and recycled strain sensors versus applied uniaxial strain.


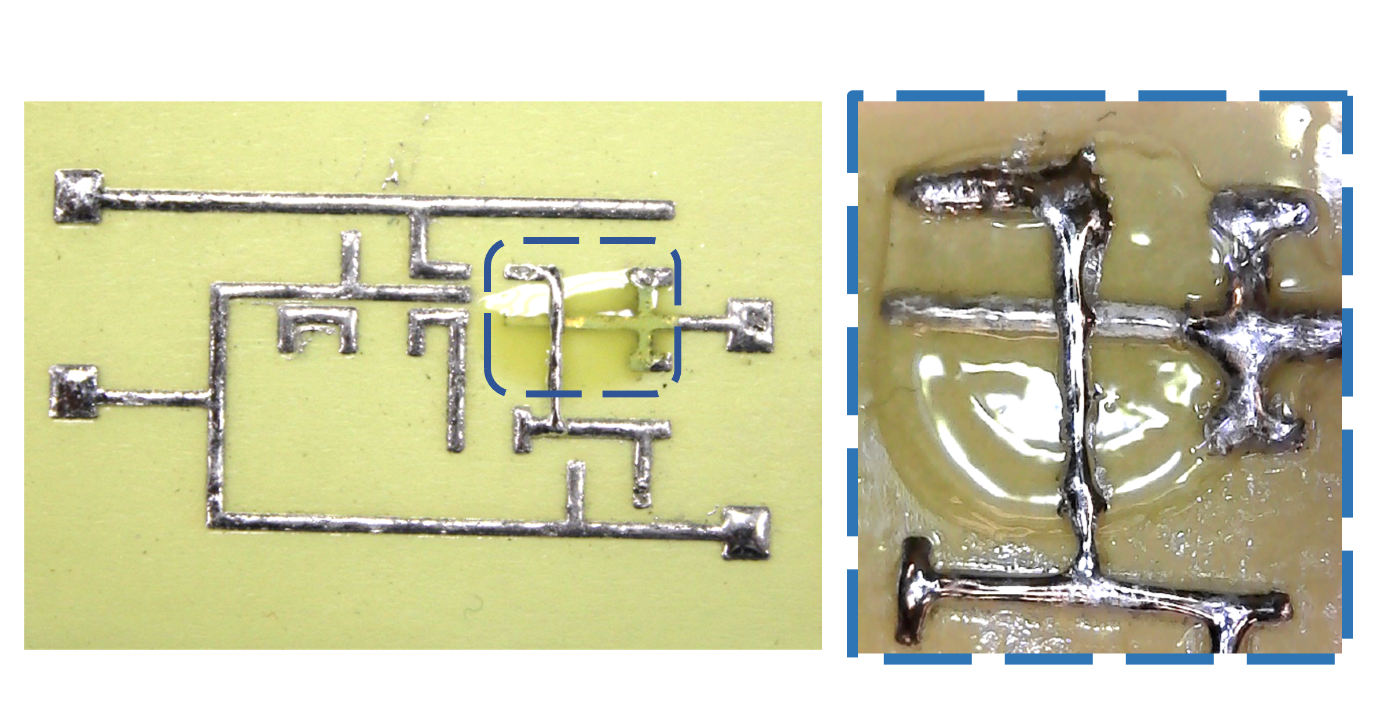


**Figure S8:** The amplifying circuit without chip components (left). Enlarged microscope image shows details of intersections of LM wires (right).


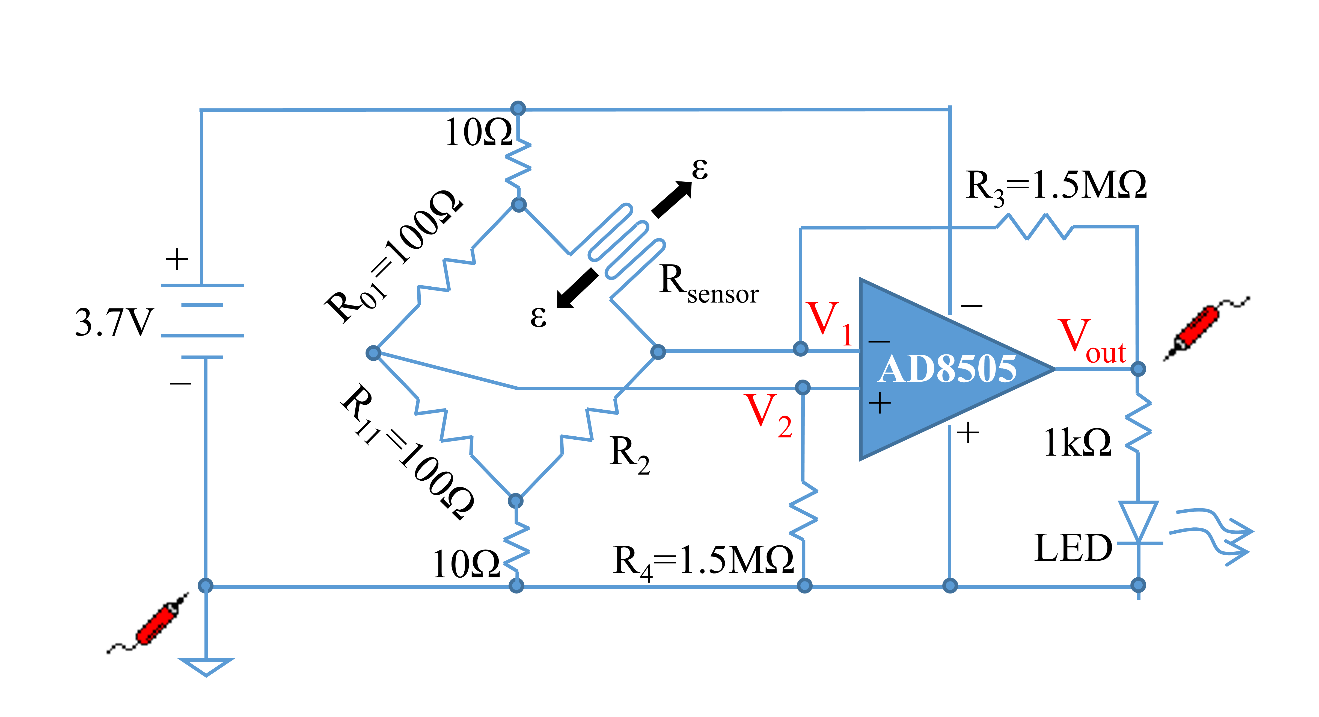


**Figure S9:** Wheatstone bridge differential amplifier circuit.


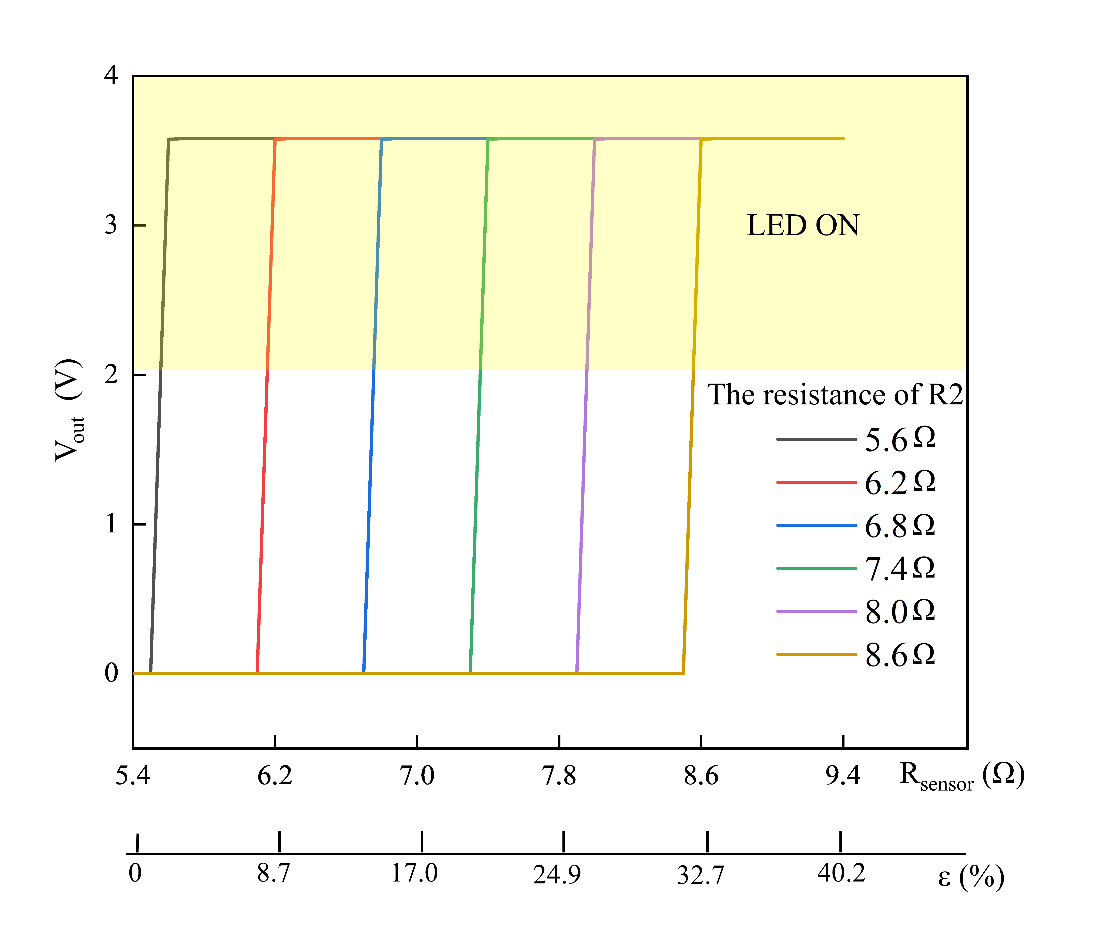


**Figure S10:** Simulated V_out_ versus R_sensor_ and the strain ε applied to the strain sensor.

**Table S1:** Performance summary of recently reported solid and liquid based stretchable strain sensors.

| Conductive material | Stretchable substrate | GF | Linearity | Strain range | Mechanical rehealability | Electrical  rehealability | reconfigurability | Recyclability | Ref. |
| --- | --- | --- | --- | --- | --- | --- | --- | --- | --- |
| AgNWs | PDMS | 14 | —— | 0~70% | No | No | No | No | [18] |
| ITO_PET | Tegaderm | 2.8 | Almost linear | 0~113% | No | No | No | No | [53] |
| GNPs/MWCNT | silicone rubber | 40.1 | nonlinear | 0~100% | No | No | No | No | [54] |
| EGaIn | Ecoflex | 0.97 | Almost linear | 0~100% | No | No | No | No | [28] |
| AuGa_2_/Ga | PDMS | 1 | nonlinear | 0~400% | No | No | No | No | [29] |
| EGaInSn | PDMS | 2.2 | Almost linear | 0~140% | No | No | No | No | [30] |
| KCl-Glycol | Ecoflex | 2.7 | R^2^=0.99 | 0~100% | No | No | No | No | [31] |
| NaCl-Glycol | Ecoflex | 3.08 | Nonlinear | 0~100% | No | No | No | No | [28] |
| Ethylene glycol | PDMS | 3.3 | Almost linear | 0~100% | No | No | No | No | [32] |
| Carbon grease ink | Ecoflex | 3.8 | Nonlinear | 0~100% | No | No | No | No | [33] |
| EGaIn-SiO_2_ | Polyimine | 2.5 | R^2^=0.995 | 0~160% | Recover ~100% | Recover ~100% | Yes | Yes | This work |
| Solid based strain sensors, and ref.53 and ref.54 used serpentine designs | | | | | | | | | |
| Liquid based strain sensors | | | | | | | | | |

**Table S2:** Performance summary of recently reported stretchable strain sensors with rehealability, recyclability or reconfigurability.

| Conductive material | Stretchable substrate | GF | Linearity | Strain range | Mechanical rehealability | Electrical  rehealability | Reconfigurability | Recyclability | Ref |
| --- | --- | --- | --- | --- | --- | --- | --- | --- | --- |
| AuNP film | sh-µAg-PU | <85 | —— | 0~35% | yes | yes | No | No | [40] |
| CNTs@(PEI@CNC) | NSCE composite | <25 | nonlinear | 0~40% | Recover ~80% | yes | No | No | [41] |
| AgNW | Hydrogel | 1.5 | —— | 0~60% | Yes | Yes | No | No | [42] |
| EGaIn | Hydrogel | 1.54 | Almost linear | 0~500% | Recover ~100% | —— | Not compatible with rehealability | No | [43] |
| SWCNT | Hydrogel | 1.51 | nonlinear | 0~1000% | —— | ~98% | No | No | [39] |
| PVA/PAANa | Hydrogel | 0.83 | —— | 0~128% | No | No | No | Yes | [50] |
| HK/PVA/NaCl | Hydrogel | 4.92 | nonlinear | 0~600% | No | No | No | Conductivity recovery ~80% | [51] |
| EGaIn-SiO_2_ | Polyimine | 2.5 | R^2^=0.995 | 0~160% | Recover ~100% | Recover ~100% | Yes | Yes | This work |
